# Snapping Biceps Femoris Syndrome: A Case Presentation and Review of Current Literature

**DOI:** 10.7759/cureus.69688

**Published:** 2024-09-18

**Authors:** Matthieu Durand-Hill, Angus Dawson, Odeh Odeh, Dominic Spicer

**Affiliations:** 1 Orthopaedics, St. Mary's Hospital, London, GBR; 2 Orthopaedics, University College London (UCL) Medical School, London, GBR

**Keywords:** biceps femoris, biceps femoris tendon snapping, bilateral fibular head, knee pain, tendon

## Abstract

Snapping of the biceps femoris tendon over the fibular head is a cause of symptomatic lateral knee pain. We presented the case of an active patient in his mid-20s who had bilateral snapping of his biceps femoris tendon, with no history of trauma. The pathophysiology in our case was thought to be secondary to prominent fibular heads. We review the literature and outline the anatomical abnormalities of the biceps femoris tendon's insertion and fibular head morphology that are thought to contribute to this condition. The pathophysiology of snapping biceps femoris tendon is likely multifactorial. Anatomical abnormalities of the biceps femoris tendon's insertion or the fibular head are thought to contribute, and correction of these abnormalities can alleviate patients' symptoms. We propose a classification system that can describe these anatomical abnormalities and guide management.

## Introduction

Snapping of the biceps femoris tendon over the fibular head is a cause of symptomatic lateral knee pain. Snapping biceps femoris syndrome, due to its poor understanding, may be underdiagnosed/underreported. Patients diagnosed with this condition will often have visited several specialists before a diagnosis is made [1].

The pathophysiologic reasons for snapping biceps femoris syndrome are grouped under anomalous tendon insertion [1-14], subluxation of an anatomically normal tendon [15,16], abnormal fibular morphology [10,17-19], or trauma resulting in a tear of the fibular attachment of the biceps femoris tendon [11,20-24]. Of these, anomalous tendon insertion is the most common cause.

In this paper, we report the case of a 24-year-old male presenting with a longstanding history of bilateral atraumatic biceps femoris snapping syndrome. In addition, we review the literature to outline the pathophysiology of biceps femoris snapping syndrome and the management options.

## Case presentation

A 24-year-old bartender attended our orthopaedic department with a two-year history of bilateral knee pain (right>left). He denied any history of trauma. Outside of work, he previously enjoyed playing regular football. However, he was unable to continue to participate due to his lateral knee pain. His only past medical history of note was Osgood-Schlatter disease.

**Figure 1:**
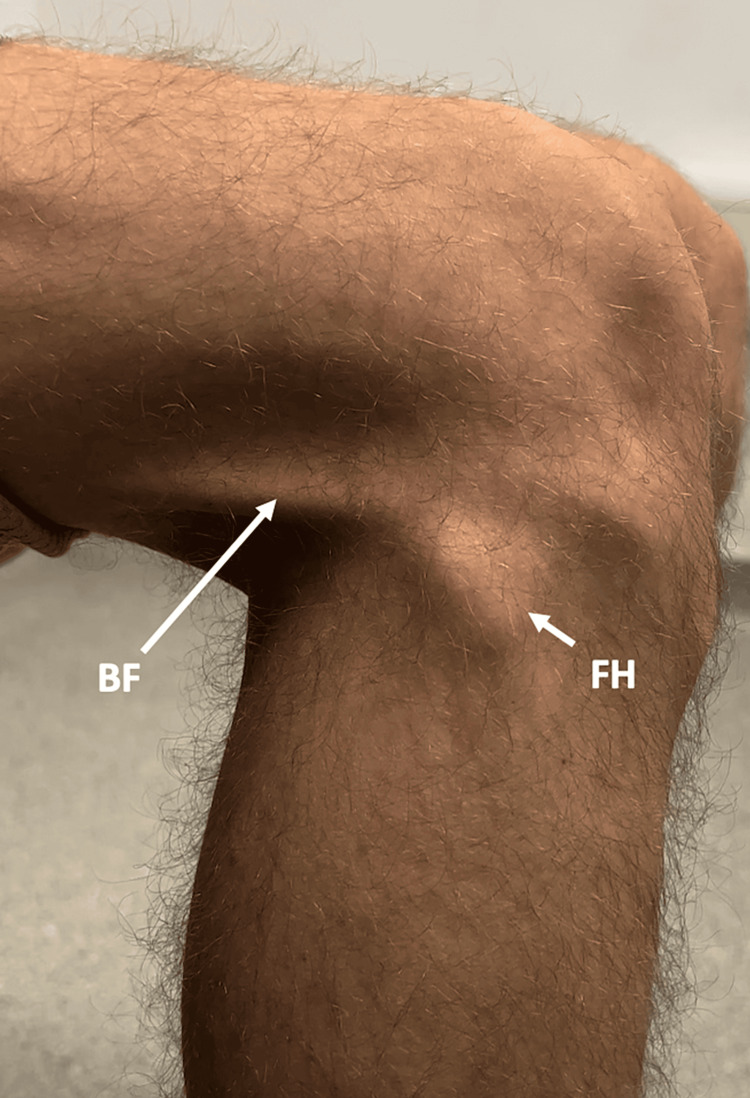
Lateral view of the patient's right knee. The biceps femoris (BF) tendon can be seen as well as a prominent fibular head (FH).

**Figure 2:**
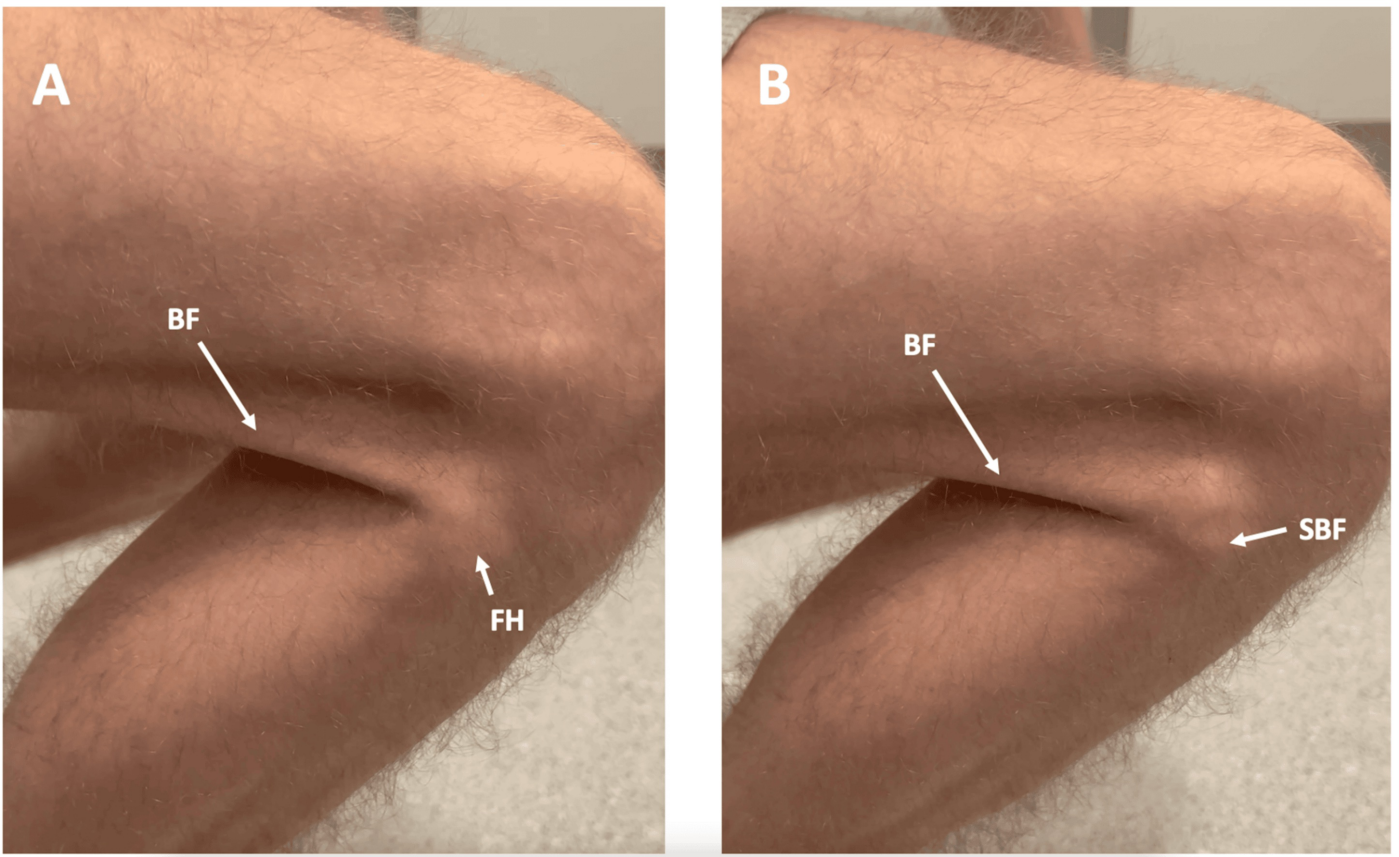
Lateral view of the patient's right knee throughout flexion. (A) The biceps femoris (BF) tendon is visualised in its normal position relative to the fibular head (FH). (B) In deep flexion, subluxation of the distal biceps femoris tendon (SBF) is visualised over the fibular head.

On clinical examination, the patient was noted to have prominent fibular heads (Figure 1). The patient had tenderness (tenderness with grimace, but no withdrawal) to palpation over the right lateral fibular head and bilaterally over the tibial tuberosities. Full active and passive range of motion was maintained, but on passive motion between 100° and 120° of flexion, the distal biceps femoris tendon was palpated and visualised to subluxate over the fibular head (Figure 2). A cracking sound was also audible, as the biceps femoris tendon subluxated over the fibular head. The patient's Musculoskeletal Health Questionnaire (MSK-HQ) score was 23/56.

Investigations

**Figure 3:**
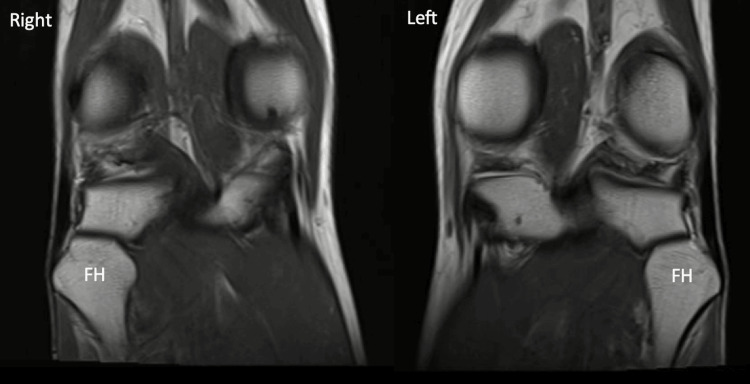
T2-weighted magnetic resonance imaging (MRI) scan of the patient's right and left knee. The prominent fibular heads (FH) are labelled and measure 30 mm in diameter.

A magnetic resonance image (Figure 3) of both knees revealed hypertrophy of the fibular head. The menisci, cruciate, collateral, and patellofemoral ligaments, iliotibial band, and extensor and popliteal tendons were unremarkable. There was no joint effusion, synovial thickening, chondral damage, or fat pad oedema. In addition, ultrasound imaging did not reveal any tendonitis of the distal biceps femoris insertion.

Treatment and follow-up

The patient is currently six months into a trial of conservative treatment. He has been referred to physiotherapy for muscle strengthening, supportive taping, bracing, and manual compression of the tendon. To date, the patient has had two sessions of in-person physiotherapy, supplemented with a home exercise programme consisting of 10-15 reps of single-leg bridges, single-leg sit-to-stands, and split squats. At the last follow-up, the patient was offered surgical intervention but opted to continue with conservative treatment. Surgical management would include debridement of the fibular head plus/minus transposition of the biceps femoris' tibial insertion to the fibular head depending on intraoperative findings.

## Discussion

There are 27 reported cases of snapping biceps femoris syndrome in the literature (Table 1). Of the 27 reported cases, only four (14.8%) patients were female. The mean age of presentation is 28 years old (with a range of 13-58 years old). Only one case previously reported was managed conservatively.

**Table 1:** Summary of current cases and classification of identified pathophysiology and treatment.

Author	Age/sex	Trauma	Bilateral	Intraoperative/radiographic findings	Treatment
Saltzman et al. [1]	13 F	No	No	Anomalous insertion of the biceps femoris to the proximal anterolateral aspect of the tibia (type 2, A2 B0)	Release of anomalous insertion and reinsertion to the fibular head
Saltzman et al. [1]	16 F	Yes	No	Anomalous insertion of the biceps femoris to the proximal anterolateral aspect of the tibia (type 2, A2 B0)	Release of anomalous insertion and reinsertion to the fibular head
Matar and Farrar [2]	49 M	No	No	Anomalous insertion of the biceps femoris tendon to the anterolateral fibula (type 1, A1 B0)	Reinsertion of the tendon to correct the position on the fibular head
Lokiec et al. [3]	23 M	No	Yes	Anomalous insertion of the biceps femoris (type 1, A1 B0)	Reinsertion of the tendon to correct the position on the fibular head
Kristensen et al. [4]	20 M	No	No	Anomalous insertion of the biceps femoris to the proximal anterolateral aspect of the tibia (type 2, A2 B0)	Resection of the tibial accessory band and partial fibular head resection
Karsen et al. [5]	36 M	Yes	No	Anomalous insertion of the biceps femoris to the proximal anterolateral aspect of the tibia (type 2, A2 B0)	Release of anomalous insertion and insertion to fibular attachment
Huang et al. [6]	58 M	No	No	Anomalous insertion of the biceps femoris tendon to the anterolateral fibula (type 1, A1 B0)	Reinsertion of the tendon to correct the position on the fibular head
Hernandez et al. [7]	16 M	Yes	Yes	Anomalous insertion of the biceps femoris into the proximal anterolateral aspect of the tibia (type 2, A2 B0)	Release of anomalous insertion and reinsertion to the fibular head
Hadeed et al. [8]	15 M	No	No	Anomalous insertion of the biceps femoris to the proximal anterolateral aspect of the tibia and the posterolateral portion of the fibula (type 2, A2 B0)	Release of anomalous insertion and insertion to fibular attachment
Guillin et al. [9]	44 M	No	No	Anomalous insertion of the biceps femoris to the proximal anterolateral aspect of the tibia (type 2, A2 B0)	-
Guillin et al. [9]	25 M	No	No	Anomalous insertion of the biceps femoris to the proximal anterolateral aspect of the tibia (type 2, A2 B0)	-
Fritsch and Mhaskar [10]	18 M	No	No	Hypertrophic femoral head and anomalous insertion of the biceps femoris with predominant tibial insertions (type 2, A2 B1)	Release of anomalous insertion from the tibia and reinsertion to the fibular head, with fibular head debridement
Fong et al. [11]	48 M	No	No	Anomalous insertion of the biceps femoris to the proximal anterolateral aspect of the tibia (type 2, A2 B0)	Release of anomalous insertion and reinsertion to the fibular head
Ernat and Galvin [12]	25 M	No	Yes	Anomalous insertion of the biceps femoris into the proximal anterolateral aspect of the tibia (type 2, A2 B0)	Bilateral anterior tenolysis
Date et al. [13]	15 M	No	No	Anomalous insertion of the biceps femoris onto the anterolateral aspect of the tibia and lateral/posterolateral aspect of the fibula (type 2, A2 B0)	Tenolysis of tibial/lateral fibular insertion
Bagchi and Grelsamer [14]	22 M	No	Yes	Prominence of the fibular head (A0 B1)	Resection of the prominent fibular head
Vavalle and Capozzi [15]	37 M	No	No	No abnormality (A0 B0)	Surgical release of tibial insertion of the biceps femoris
Crow et al. [16]	49 M	No	No	No abnormality (A0 B0)	Surgical release of tibial insertion of the biceps femoris
Padovani et al. [17]	21 M	Yes	No	Torn fibular insertion and prominence of the fibular head (traumatic A2 B1)	Reattachment of the torn tendon to the fibular head. Debridement of the prominent lateral fibular head
Bernhardson and LaPrade [18]	28 M	Yes	No	Torn fibular insertion (traumatic A3 B0)	Reattachment of the torn tendon to the fibular head
Bernhardson and LaPrade [18]	43 F	No	No	Torn fibular insertion (traumatic A2 B0)	Reattachment of the torn tendon to the fibular head
Bernhardson and LaPrade [18]	41 F	No	No	Torn fibular insertion (traumatic A2 B0)	Reattachment of the torn tendon to the fibular head
Bansal et al. [19]	19 M	Yes	No	Torn fibular insertion (traumatic A2 B0)	Reattachment of the torn tendon to the fibular head
Reid and Mofidi [20]	15 M	No	Yes	Prominence of the fibular head. Anomalous insertion of the biceps femoris to the proximal anterolateral aspect of the tibia (A2 B1)	Reattachment of tibial insertion to the fibular head
Zadeh et al. [21]	19 M	No	Yes	Prominence of the fibular head (A0 B1)	Conservative
Bach and Minihane [22]	24 M	No	Yes	Prominence of the fibular head (A0 B1)	Resection of the prominent fibular head
Fung et al. [25]	17 M	No	Yes	Exostosis of the fibular head (A0 B2)	Resection of exostosis

The biceps femoris tendon serves as a crucial dynamic stabiliser of the knee, contributing to the prevention of both anterolateral and anteromedial instability. Despite its importance, the precise anatomical insertion of the biceps femoris tendon has traditionally been oversimplified, as a single insertion onto the fibular head [21]. However, contemporary anatomical studies have revealed the biceps femoris insertion to be more complex. Tubbs et al. [23] dissected 56 cadavers and revealed that the distal biceps femoris tendon is divided into medial and lateral slips by the lateral collateral ligament. Each of these slips is then further divided into the anterior and posterior components. The posterior components insert onto the fibular head and the anterior components insert onto the proximal lateral tibia, popliteus tendon, and arcuate ligament.

The most common pathophysiological reason for snapping biceps femoris syndrome reported in the literature is an abnormality of the tendon's distal insertion. Catonné et al. [24] classified the abnormal biceps femoris insertions into three types: type 1, predominant fibular arm and accessory tibial arm and insertion excessively anterior on the fibular head; type 2, predominant tibial arm and fibular arm present but accessory; and type 3, tibial arm only. Although 80% of cases with this pathophysiological mechanism were reported as unilateral, it is likely the abnormal insertion is bilateral. However, the asymptomatic side is typically not imaged. Furthermore, in a magnetic resonance imaging (MRI) study of 403 asymptomatic patients, 10% were found to have predominant tibial insertions (type 2), and 1% were found to have exclusive tibial insertions (type 3). This suggests that the abnormal insertion may predispose an individual to a snapping biceps femoris tendon, but its presence alone does not always manifest as snapping.

Another cause of snapping biceps femoris syndrome is trauma resulting in a tearing of the biceps femoris' fibular insertion. Within these patients, their normal distal biceps femoris insertion is essentially converted into a type 2/3 insertional abnormality depending on the extent of the injury, with conversion to a type 3 insertional abnormality occurring in patients with complete rupture of the fibular insertion. These patients have successfully been managed surgically with reinsertion of the torn biceps femoris to the fibular head or rerouting the tibial insertion to the fibular head.

A prominent fibular head was found in seven (25.9%) of the previously reported cases, either in isolation (four cases) or in combination with tendinosis (one case) or an abnormal tendon insertion (two cases) [11,14,17,20-22]. The majority of these cases (71.4%, n=5) were bilateral, as was the case in our patient. Management of symptomatic patients with a prominent fibular head either was conservative (n=1) or involved resection/debridement of the fibular head in combination with tendon transposition [25].

**Figure 4:**
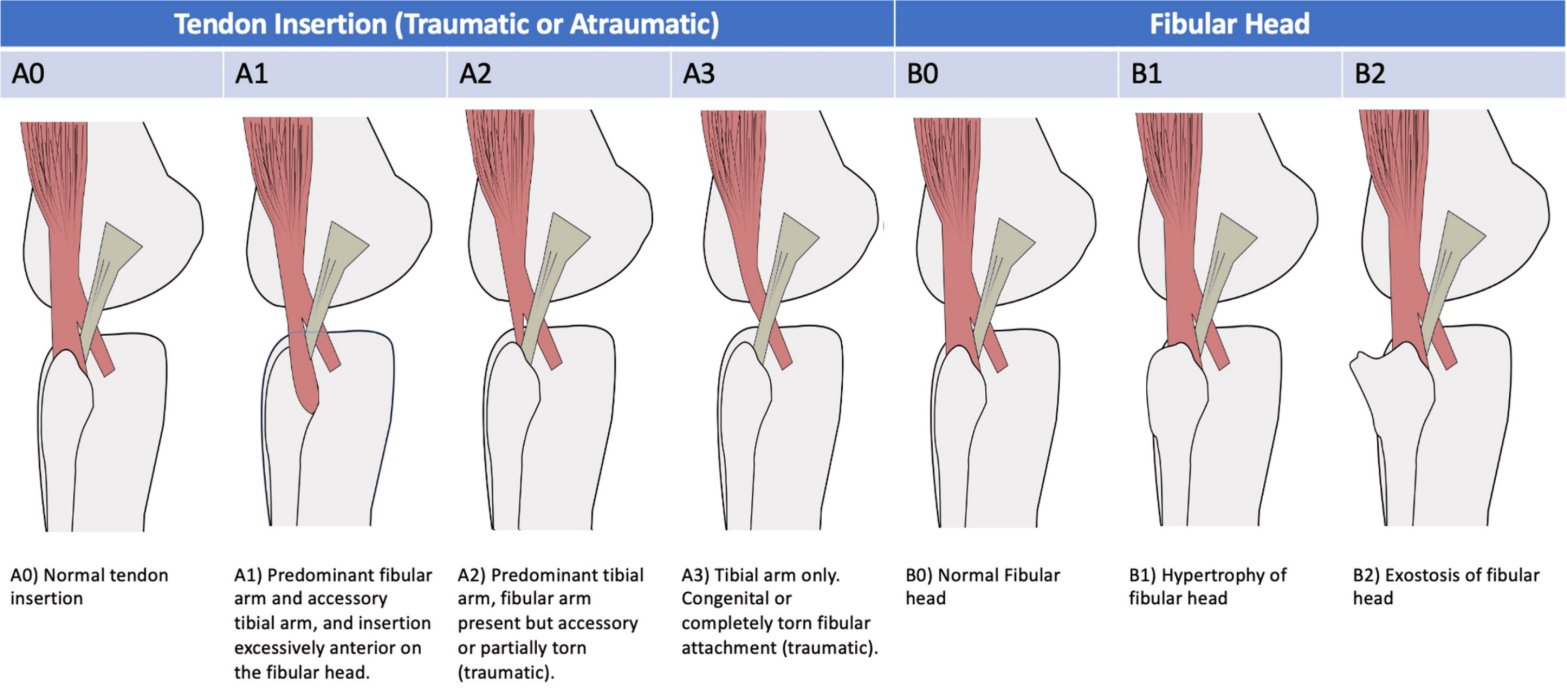
Proposed classification of the anatomical abnormalities in patients with snapping biceps femoris syndrome. Image Credit: Matthieu Durand-Hill

We propose a two-part classification system (Figure 4) to help understand the anatomical abnormalities that contribute to snapping biceps femoris syndrome and guide management. The majority of cases are caused by an abnormality either of the tendon insertion, which may be traumatic or congenital in origin, or of the fibular head (hypertrophy/bursitis or an exostosis). Management options include conservative or surgical. Conservative management is with non-steroidal anti-inflammatories and physiotherapy and should be trialed initially. Surgical correction depends on the pathophysiology. Patients with an A1 component may be treated with reinsertion of the excessively anterior fibular head more posteriorly. Patients with atraumatic A2/A3 insertions may be treated with the release of the anomalous insertion from the tibia and reinsertion to the fibular head. If trauma has resulted in an A2/A3 insertional pattern, then the torn fibular component may be repaired, or the tibial insertion may be transposed to the fibular head. Patients with an abnormal fibular head morphology (B1 or B2) will typically require fibular head debridement.

## Conclusions

The pathophysiology of snapping biceps femoris tendon is likely multifactorial. Within the literature, anatomical abnormalities of the fibular head or tendon insertion are thought to contribute, either together or in isolation. Three separate insertional abnormalities of the biceps femoris have been described in the literature. These can be traumatic or atraumatic. We propose a classification system that can describe these anatomical abnormalities and guide management. Management of this condition may be conservative or surgical. Surgical options include resection of a prominent fibular head, reattachment of the biceps femoris to the fibular head, or surgical release of the biceps femoris' tibial insertion.
